# Cerebellum: An explanation for dystonia?

**DOI:** 10.1186/s40673-017-0064-8

**Published:** 2017-05-12

**Authors:** Matteo Bologna, Alfredo Berardelli

**Affiliations:** 1 Department of Neurology and Psychiatry and Neuromed Institute, Sapienza University of Rome, Viale dell’Università, 30, 00185 Rome, Italy; 2 Neuromed Institute IRCCS, Pozzilli, IS Italy

**Keywords:** Dystonia, Cerebellum, Basal ganglia, Pathophysiology, Motor control

## Abstract

Dystonia is a movement disorder that is characterized by involuntary muscle contractions, abnormal movements and postures, as well as by non-motor symptoms, and is due to abnormalities in different brain areas. In this article, we focus on the growing number of experimental studies aimed at explaining the pathophysiological role of the cerebellum in dystonia. Lastly, we highlight gaps in current knowledge and issues that future research studies should focus on as well as some of the potential applications of this research avenue. Clarifying the pathophysiological role of cerebellum in dystonia is an important concern given the increasing availability of invasive and non-invasive stimulation techniques and their potential therapeutic role in this condition.

## Background

The number of investigations on the pathophysiology of dystonia has grown considerably over the past 40 years. Research in the field of dystonia pathophysiology, however, is faced with a number of problems. One major problem is that dystonia is etiologically and phenomenologically heterogeneous [1–4]. The majority of dystonia cases are idiopathic and a small proportion of patients have a positive family history of dystonia, or also have other movement disorders. In idiopathic dystonia, no obvious neuronal degeneration is detected and it is difficult to identify a primary defect. In secondary cases, the location of structural lesions may provide some insight into the neural structures involved in the pathophysiology of dystonia. However, observations in secondary dystonia cases may not always be easily generalizable to idiopathic forms. Yet another problem is the variable body distribution of the motor symptoms of dystonia, which may range from isolated involvement of one body segment in blepharospasm (BPS), oromandibular dystonia (OMD), cervical dystonia (CD), focal-hand dystonia (FHD) and task-specific dystonia (writer’s cramp or embouchure dystonia) to more generalized involvement of several body regions [1–4]. Thus, putative pathophysiological mechanisms in one condition may not be directly applicable to other dystonia forms. Lastly, important information has been yielded by animal models, in which evidence is usually strong and highly reliable [5–7]. Species differences, however, raise the question whether the results of animal studies are useful for the understanding of dystonia pathophysiology in patients [8]. Also, differently from studies on animals, experimental studies on dystonia patients are, with the exception of the analysis of basal ganglia field potentials recorded from implanted deep brain stimulation (DBS) electrodes in the globus pallidus internus [9], based on non-invasive techniques, including neurophysiology and neuroimaging, which only allow indirect inferences to be made on the underlying pathophysiological mechanism. For all the aforementioned reasons, the major and still unsolved issue in human patients concerns the distinction between different types of causality, i.e. the attribution of mechanisms that are specifically linked to the development of dystonia, to compensation or to an epiphenomenal association [10, 11].

Several lines of evidence suggest that the basal ganglia may play a causative role in dystonia [12]. Experimental studies on animals indicate that several basal ganglia abnormalities and neuronal activity throughout specific cortico-subcortical loops are involved in the expression of dystonia [13]. Clinical observations demonstrate that dystonia is the movement disorder most frequently associated with structural lesions of the basal ganglia [14, 15]. DBS recordings show that dystonia occurs as a result of disrupted activity of the basal ganglia thalamic circuit, including altered firing patterns, synchronized oscillations and wider receptive fields [9, 16, 17]. Lastly, neurophysiological studies on dystonia patients have provided evidence of major abnormalities, such as reduced inhibition, maladaptive plasticity and altered sensory processing, that indirectly reflect the influence of altered basal ganglia on cortical areas and the brainstem [1, 18–21]. It is noteworthy that these abnormalities are often independent of dystonic symptoms, i.e. they are detected in unaffected body parts, or in unaffected relatives of patients, which may point to an endo-phenotypic predisposition, i.e. the substrate on which the dystonia develops, and a close causative relationship with the disease [1, 20, 21].

More recent studies have also indicated that dystonia is a network disorder due to abnormalities not only in basal ganglia but also in other interconnected structures, including the cerebellum and the cortical areas [8, 10, 11, 13, 22–25]. However, the pathophysiological role of cerebellum in dystonia has yet to be fully understood. In this article, we present an updated review, based on data from animals, clinical and pathological observations as well as neurophysiological and neuroimaging evidence in human patients, of the increasing body of literature suggesting a pathophysiological role of cerebellum in this condition. We will also highlight gaps in current knowledge and issues that future research studies should focus on.

## Review

### Animal studies

Experimental studies on animals have consistently shown that abnormal cerebellar signalling may produce dystonia-like movements. Pharmacological excitation of the cerebellar cortex using local microinjections of kainic acid in normal mice elicits reliable and reproducible dystonic postures of the trunk and limbs [26, 27]. The severity of dystonia usually increases linearly with the kainate dose. Conversely, kainate-induced dystonia is reversed or reduced by the glutamatergic antagonist [26]. Furthermore, in transgenic mice lacking Purkinje cells, kainic acid injections induce a significantly lower degree of dystonia, thus pointing to a role of the major output cells of the cerebellum in generating dystonic movements [26].

When dystonic movements are triggered by pharmacological stimulation of the cerebellum, microdialysis reveals significant reductions in striatal dopamine release [27]. Chronic application of kainic acid into the cerebellar vermis of rats results in a prolonged and generalized dystonia motor phenotype and increased c-fos expression, a marker of neuronal activation, not only in the cerebellum but also in other structures, including the ventral-anterior thalamus [28].

Another animal model of dystonia is that of tottering mice, which exhibit paroxysmal dystonia due to an inherited defect affecting calcium channels. Surgical removal of the cerebellum abolishes the dystonic attacks in the tottering mice model while subclinical striatal lesions exaggerate their generalized dystonia [27].

By using conditional genetic manipulations to regionally limit cerebellar dysfunction, Raike et al. [29] observed that the abnormalities restricted to Purkinje cells cause dystonia and that the extent of cerebellar dysfunction determines the severity of abnormal movements. Dysfunction of the entire cerebellum causes abnormal postures in many body parts that resemble generalized dystonia. More limited regions of cerebellar dysfunction produce abnormal movements in an isolated body part that resemble focal dystonia. Vanni et al. [30] also found that a loss of function of torsinA, a ubiquitous protein with peculiar developmental expression in striatum and cerebellum [31], leads to widespread alterations of cerebellar synaptogenesis that might contribute to the age-dependent susceptibility to dystonia in mutation carriers. More recently, torsinA was knocked down in the cerebellum and in the basal ganglia of adult mice. TorsinA knockdown in the cerebellum is able to induce an irregular cerebellar output, i.e. changes in the intrinsic activity of both Purkinje cells and neurons of the deep cerebellar nuclei and to induce dystonia. These data point to the cerebellum as the main site of dysfunction in DYT1 [32].

In summary, data from studies on animals suggest an important role of the cerebellum as well as altered functional relationships between cerebellar and basal ganglia circuits in generating dystonic symptoms, thereby supporting the assumption that a disturbed neuronal network is involved in the pathophysiology of this disorder. Abnormal motor symptoms are associated with irregular cerebellar output caused by changes in the intrinsic activity of both Purkinje cells and neurons of the deep cerebellar nuclei. Interestingly, recent data support the hypothesis that a dysfunction of the whole cerebellum causes abnormal movements and postures in many body parts, thus resembling generalized dystonia, whereas a more limited cerebellar dysfunction causes dystonia in an isolated body part.

### Human studies

#### Clinical and pathological observations

Several case series and case reports indicate a relationship between anatomical lesions of the cerebellum and dystonia [33–53], (Table 1). These studies indicate that majority of secondary cases of CD are associated structural lesions of the brainstem and cerebellum, with lesions in the cervical spinal cord and basal ganglia being observed less frequently [33–43]. Despite being inconsistent, head rotation tended to be contralateral to the side of the lesion. The relative lack of damage in the basal ganglia combined with the concentration of lesions in the brainstem and cerebellum and in patients with secondary CD suggests that cerebellar afferent pathway dysfunction may also play an important role in the pathophysiology of primary CD [34].

**Table 1:** Major studies on the association between the anatomical lesions of the cerebellum and dystonia

[ref n°] Study	Type of study	Major findings: type of dystonia/lesion
[33] Boisen,1979	Case series	CD/infra-tentorial tumors
[49] Rumbach et al.,1995	Case report	Hemidystonia/extracranial vertebral artery dissection and cerebellar infarction
[35] Caress e al.,1996	Case report	CD/cerebellar gangliocytoma
[50] Ghika-Schmid et al.,1997	Case series	Focal and segmental dystonia involving the upper limbs/cerebellar strokes
[36]Krauss et al., 1997	Case series	CD/posterior fossa tumors
[51] Alarcon et al., 2001	Case report	upper limb dystonia/tuberculoma in the ipsilateral cerebellar hemisphere
[34] LeDoux and Brady 2003	Case series	CD/lesions of the cerebellum and of its primary afferent pathways
[37] Kumandaş et al., 2006	Case series	CD/spinal or posterior fossa tumors
[44] LeBer et al., 2006	Case series	Focal, multifocal and generalized dystonia/cerebellar atrophy
[38] O’Rourke et al., 2006	Case report	Paroxysmal CD and BPS/bilateral cerebellar infarction
[39] Zadro et al., 2008	Case report	CD/cerebellar stroke
[46] Alcalay et al., 2009	Case report	OMD/cholestanol deposition in the in the dentate nuclei (cerebrotendinous xanthomatosis)
[47] Waln and LeDoux 2010	Case report	OMD/hemorrhage of the vermal and paravermal regions of the cerebellum
[40] Usmani et al. 2011	Case report	CD/haemorrhage of the cerebellar vermis
[52] Baik et al., 2012	Case report	upper limb dystonia/isolated cerebellar hemiatrophy
[41] Kojovic et al., 2012	Case report	CD/cystic lesion in the right cerebellar hemisphere
[45] Khooshnoodi et al. 2013	Case series	BPS/ischemic stroke in the cerebellar hemisphere and in the inferior cerebellar peduncle, cystic lesion in the dentate nucleus
[42] Fąfara-Leś et al., 2014	Case series	CD/posterior fossa tumours
[43] Batla et al., 2015	Case series	CCD/cerebellar atrophy or lesions
[48] Bana et al., 2015	Case report	OMD/haemorrhage of the vermian and left paramedian regions of the cerebellum

The various studies are listed in chronological order. Cervical dystonia (CD); cranio-cervical dystonia (CCD); blepharospasm (BPS); oromandibular dystonia (OMD)

Batla et al. [43] retrospectively reviewed a large series of patients with cervical/segmental dystonia; a significant proportion of the patients displayed signs of cerebellar atrophy or other abnormalities on neuroimaging, including low-grade tumor, cerebellar infarct cyst, white matter hyperintensity and ectopia. However, owing to the retrospective nature of the study, it is difficult to ascertain a possible relationship between the location of the cerebellar abnormalities and the clinical distribution of dystonia. LeBer et al. [44] described 12 patients from eight families with an unusual dystonia-plus phenotype characterized by focal or multifocal dystonia associated with scarce clinical cerebellar signs but marked cerebellar atrophy on neuroimaging in most patients. Interestingly, dystonia became generalized in a significant proportion of the patients.

The relationship between cerebellar lesions and clinical manifestations of dystonia is supported by further observations in secondary BPS and OMD and in patients with upper-limb dystonia often ipsilateral to a focal cerebellar lesion [45–53]. Also, it has been recently observed patchy loss of cerebellar Purkinje cells in the cerebellum in a small sample patients with CD. The latter observations, however, require confirmation in a large sample of clinically well characterized patients with dystonia [54].

#### Neurophysiological studies

##### Transcranial magnetic stimulation (TMS)

TMS is one of the most widely adopted neurophysiological techniques in human studies [55–61]. TMS techniques can be used to stimulate the cerebellum system and to investigate various physiological mechanisms, including connectivity measures and plasticity of the cerebello-thalamo-cortical pathways, in both physiological and pathological conditions, including dystonia [62, 63].

Paired-TMS techniques, which deliver stimuli over the cerebellum followed, at various interstimulus intervals, by stimuli over the contralateral primary motor cortex (M1), can be used to investigate connectivity between the cerebellum and M1 [64]. A number of studies have shown that repetitive TMS (rTMS) stimuli, i.e. 1 Hz rTMS and theta burst stimulation (TBS), induce bidirectional (i.e. increase or decrease) changes in MEP amplitude, as assessed by single-pulse TMS over the contralateral M1 [65–69]. These changes in MEP amplitude are believed to be an implicit measure of an underlying plasticity mechanism, i.e. long-term potentiation and long-term depression-like effects within the cerebello-thalamo-cortical pathways. Studies have shown that 1 Hz rTMS applied over the cerebellum has a modulatory effect on interconnected motor areas and increases MEP amplitude, thereby suggesting that Purkinje output to the dentate nucleus is reduced, which would, in turn, be accompanied by dentate-cortical drive disinhibition [64–66]. The effects of cerebellar iTBS or cTBS are, unlike those of 1 Hz rTMS, likely to be exerted on the superficial layer of the cerebellar cortex (which inhibits Purkinje cell activity). As a consequence, cerebellar iTBS or cTBS inhibits or facilitates Purkinje cells (inhibitory output) and the dentate-thalamo-cortical pathway [67, 69].

Studies based on different TMS techniques have assessed the influence of cerebellar single-pulse TMS or cTBS on M1 excitability in FHD patients. Overall, these studies indicate that a conditioning cerebellar stimulus [70] or cerebellar cTBS [71, 72] does not induce any inhibitory effect on the contralateral M1 in patients with FHD. The lack of any inhibitory cerebellar effect on the contralateral M1 in FHD patients may contribute to the loss of M1 inhibition, to the maladaptive sensorimotor plasticity of M1 and to the emergence of incorrect motor programs and adaptive behaviours [71, 72]. Alternatively, reduced cerebellar inhibitory modulation of M1 in FHD might reflect compensatory changes in this disorder [71, 72].

TMS has also rarely been used to assess the influence of cerebellar single-pulse TMS or cTBS on M1 excitability in other dystonia subtypes. We recently compared the effect of cerebellar cTBS on M1 excitability in FHD and CD patients. We found that cerebellar cTBS inhibits M1 excitability in CD patients though not in FHD patients [72].

In summary, the results of TMS studies on patients with dystonia indicate that the cerebellum may affect M1 in different forms of focal dystonia in various ways. The involvement of the cerebello-thalamo-cortical pathways may be a feature of FHD but not of other dystonia types.

##### Movement studies, adaptive motor learning and motor time processing

The cerebellum contributes to sensorimotor integration and regulates the coordination of voluntary movements through neural connections with M1 [73, 74]. Thus, cerebellar dysfunction leads to a range of voluntary movement abnormalities, including slowness, impaired timing, overshooting/undershooting and increased curvature of movement trajectories [74, 75].

A number of studies indicate movement slowness in patients with primary focal dystonia [76–78]. There is also evidence showing that reaching movements are impaired in patients with idiopathic dystonia involving the upper limb and other body segments [79] as well as in patients with CD [80, 81]. Nowak et al., [82] also investigated the kinematic features of reaching-to-grasp-movements in patients with BPS and CD and found prolonged movement times and lower peak velocities of hand transport in patients than in healthy subjects. However, peak grasp aperture and the timing of peak grasp aperture in relation to the time of hand transport did not differ between patients and healthy subjects, reflecting the accurate sensorimotor integration [82]. More recently Katschnig-Winter et al. [83] found that upper limb motor performance in patients with CD is similar to that in healthy controls, with the exception of significantly higher peak velocities in patients. Similarly, we also found that reaching movements are normal in both FHD and CD patients, not only in terms of duration, velocity and acceleration but also in terms of the quality of the movement, curvature of movement trajectory, smoothness of velocity curves and target overshooting [72].

Motor sequence learning and motor adaptation, i.e. the process of adjusting movement to new environmental demands, rely on partially overlapping circuits in major brain regions, i.e. M1, the basal ganglia and cerebellum [84, 85]. Abnormalities in motor sequence learning have been observed in non-manifesting carriers of the DYT1 dystonia mutation [86, 87]. In focal dystonia, however, Katschnig-Winter et al. [83] observed that CD patients and healthy controls had similar levels of motor sequence learning and motor adaptation. Sadnicka et al. [88] used a visuomotor task to test the hypothesis that cerebellar abnormalities in CD patients translate into motor adaptation deficits. However, not only were adaptation rates (learning) in CD patients found to be similar to those of healthy subjects, but the adaptive ability was not related to the clinical features of CD, thus going against the hypothesis that adaptive learning abnormalities reflect cerebellar dysfunction in CD. It has also recently been reported that patients with BPS and writer’s cramp are significantly impaired on various adaptation walking parameters whereas walking parameter adaptation in CD patients, including speed, step width, step length symmetry and swing/stance ratio, does not differ from that in healthy controls [89].

Motor time processing is another important function for several cognitive tasks and motor activities, mediated by a neural network including cerebellum basal ganglia and cortical areas [90–92]. Patients with writer’s cramp or CD less accurately predict the temporal outcome of a visually perceived movement. These data suggest that patients with dystonia manifest subtle deficits in extrapolating temporal properties during the perception of hand/arm movements [91, 92]. Patients with CD also exhibit impaired performance on a motor time estimation tasks, which requires proper temporal prediction and duration estimation; these findings thus provide further evidence on altered cerebellar function and altered interactions between the cerebellum and the basal ganglia in dystonia [93, 94].

Taken together, the results suggest that integration of proprioceptive input, which is involved in the internal models of limb dynamics, may be altered in FHD, whereas data in CD are more contrasting. A plausible explanation is that the cerebellar representation of the hand muscles prevails over that of the axial muscles, including the neck muscles [95]. Finally, the results point to variable adaptive learning abnormalities and to a deficit in motor time processing in different dystonia types.

##### Eyeblink classical conditioning (EBCC)

EBCC is a cerebellum-dependent paradigm of associative motor learning, and abnormal EBCC is a neurophysiological indicator of cerebellar dysfunction [96]. Studies based on the EBCC paradigm have shown that cerebellar associative learning is impaired in patients with adult onset primary focal dystonia, i.e. FHD and CD [97, 98]. The response to cerebellar conditioning, as assessed by the EBCC, has also been shown to be abnormal in myoclonus-dystonia patients [99]. It is, however, unclear whether the impaired EBCC observed in patients with primary dystonia is due to actual cerebellar pathology or reflects functional cerebellar disruption. Hoffland et al. [100] observed that cerebellar cTBS paradoxically normalized EBCC in patients with CD. These findings point to a functional and reversible disruption of the cerebellum in dystonia, a phenomenon that is likely to be secondary to either cerebellar compensation or to cerebellar recruitment in the abnormal sensorimotor network.

However, the response to the EBCC paradigm in patients with secondary dystonia does not differ from that in healthy controls [98]. More recent EBCC-based investigations in patients with DYT1 and DYT6 dystonia have also shown that these patients’ ability to acquire conditioned responses is normal [101].

Patients with dystonic tremor display a decreased number of conditioned responses in the EBCC paradigm than either dystonic patients without tremor or HS. Data show that cerebellar impairment segregates with the presence of tremor in patients with dystonia, thus suggesting that the cerebellum might play a specific role in the occurrence of dystonic tremor [102].

To sum up, studies based on EBCC suggest that cerebellar abnormalities may play distinct roles in different subsets of dystonia and be related to specific symptoms, including tremor, as opposed to playing a primary role in generating dystonic symptoms.

#### Neuroimaging

Numerous structural, functional and molecular imaging technique have been applied in dystonia patients and several abnormalities have been reported in the sensorimotor cortex, basal ganglia and cerebellum [103]. In some cases, structural neuroimaging studies have reported prominent grey matter (GM) changes involving the infratentorial areas in patients with primary dystonia compared with healthy controls [104], however, a clear pattern of GM alteration has not been established in dystonia [105]. Studies based on fractional anisotropy (FA), a measure of axonal integrity, have also shown that patients with CD have lower FA values in the genu and in the body of the corpus callosum and lower MD values in the left pallidum, the left putamen and the caudate bilaterally, without displaying any evidence of cerebellar involvement [106]. Carbon et al. [107] that FA was significantly reduced in primary dystonia patients in the pontine brainstem in the vicinity of the left superior cerebellar peduncle and bilaterally in the white matter of the sensorimotor region.

Recent evidence has shown significantly lower resting-state functional connectivity, involving the cerebellum, thalamus, basal ganglia and frontal areas, in patients than in healthy subjects [108]. Abnormal resting-state functional connectivity of sensorimotor representations of affected and unaffected body parts points to a pathophysiological predisposition for abnormal sensorimotor and audio-motor integration in embouchure dystonia [109]. When Prudente et al. [110] explored the neural substrates for head movements in subjects with CD, they found that isometric head rotation in the direction of dystonia was associated with greater activation in the ipsilateral anterior cerebellum, which suggests that cerebellum involvement in specific areas contributes to the abnormal head rotation in CD. Event-related functional magnetic resonance imaging, combined with tactile stimulation of dystonic (upper lip) and non-dystonic (forehead and dorsal hand) body regions, has shown that musicians with embouchure dystonia display increased stimulation-induced activity in contralateral primary and bilateral secondary somatosensory representations of dystonic and non-dystonic body regions as well as in the cerebellum ipsilateral to the left dystonic upper lip [111].

Studies on resting regional metabolism have revealed consistent abnormalities in primary dystonia involving multiple interconnected elements of these circuits. In gene carriers, changes in specific subsets of these regions have been found to relate to genotype, phenotype, or both [112].

In summary, a range of neuroimaging techniques has been used in patients with dystonia over the last decade. Structural, functional and molecular imaging techniques have revealed abnormalities, above all in the sensorimotor cortex, basal ganglia and cerebellum. The results of in vivo neuroimaging studies, most of which have been structural, show that subtle morphological abnormalities occur in idiopathic dystonia. These data challenge the concept that no brain structure abnormalities are present in patients with idiopathic dystonia and that this movement disorder is solely due to abnormal cerebral function. Although no clear pattern of brain structural abnormality has yet emerged in dystonia, functional MRI techniques have revealed a closer relationship between areas of cerebellar involvement and the corresponding body areas affected by dystonia.

### Future research issues

The cerebellum is now widely considered to play an important role in cognition and other non-motor functions [74]. In addition, a growing body of evidence suggests that an important non-motor component is involved in primary dystonia, including neuropsychiatric, cognitive, sleep and sensory function abnormalities [113, 114]. Although the relationship between cerebellar abnormalities and non-motor symptoms in dystonia has not yet been assessed, altered cerebellar activity in dystonia is likely to play a role in generating non-motor symptoms in dystonia patients and deserves further investigation to shed light on the pathophysiology of dystonia.

Botulinum toxin type A (BoNT-A) weakens muscle contraction by inhibiting peripheral acetylcholine release from the presynaptic neuromuscular terminals. In addition, BoNT-A alters sensory input to the central nervous system, thus indirectly inducing secondary central changes. Some of the long-term clinical benefits of BoNT-A treatment may also reflect changes in spinal, brainstem and cortical circuits, including cortical excitability and plasticity/organisation, by altering spindle afferent inflow directed to spinal motoneurons or to the various cortical areas in dystonia [115–117]. Future research should focus on clarifying the relationship between cerebellar changes in dystonia and the resolution of these disease-related functional abnormalities following treatment.

Recent studies on animals point the cerebellum as s major site of dysfunction in DYT1 dystonia [48]. Nevertheless, evidence in human (manifesting or non-manifesting) carriers of the DYT1 mutation is still limited. Moreover, neurophysiological studies on altered motor sequence learning [86, 87] but normal EBCC [101] or neuroimaging investigations [112] in DYT1 mutation carriers have provided heterogeneous results. Future studies should better delineate to what extent evidence of cerebellar abnormalities in DYT1 mutation carriers relates to the phenotypic variability of dystonia [118, 119]. The results of these studies might have relevant clinical implication, with regard to disease severity and age of onset prediction.

### Conclusions

Several lines of evidence indicate that dystonia may result from basal ganglia abnormalities. The suggestion that the cerebellum plays a pathophysiological role in dystonia is based on a growing number of animal studies as well as on clinico-pathological observations and experimental investigations in patients using various neurophysiological and neuroimaging techniques. Either the dysfunction of basal ganglia and cerebellum or an abnormal interaction between these two systems may vary with different etiologies of dystonia supporting the hypothesis of differing pathophysiology in different forms, i.e. primary or secondary dystonia or generalized and focal dystonia. Unlike the M1 plasticity mechanism or sensory processing abnormalities, which are likely to result from basal ganglia dysfunction and are present throughout the cortical sensorimotor system, and even in unaffected relatives of dystonia patients, abnormal cerebellar effects are mainly found in cortical areas that control the affected body parts. Thus, existing experimental evidence suggests that cerebellar dysfunction is likely to be related to body areas affected by dystonia as opposed to being a widespread (i.e. endo-phenotypic predisposition) pathophysiological abnormality of the disease, which means the cerebellar involvement in dystonia may affect the topographic expression of dystonic symptoms. Future studies on dystonia should focus on the pathophysiological role of the cerebellum and on its abnormal interaction with the basal ganglia. Clarifying this issue is relevant because of the increasing availability of invasive and non-invasive stimulation techniques and their potential therapeutic role in dystonia.

## Abbreviations

BPS: Blepharospasm
CD: Cervical dystonia
DBS: Deep brain stimulation
EBCC: Eyeblink classical conditioning
FHD: Focal-hand dystonia
GM: Grey matter
M1: Primary motor cortex
MEP: Motor evoked potentials
OMD: Oromandibular dystonia
TBS: Theta burst stimulation
TMS: Transcranial magnetic stimulation
